# Psychometric properties, factor structure, and German population norms of the multidimensional fatigue inventory (MFI-20)

**DOI:** 10.3389/fpsyt.2022.1062426

**Published:** 2022-12-20

**Authors:** Adrian Westenberger, Mariel Nöhre, Elmar Brähler, Matthias Morfeld, Martina de Zwaan

**Affiliations:** ^1^Department of Psychosomatic Medicine and Psychotherapy, Hannover Medical School, Hanover, Germany; ^2^Integrated Research and Treatment Center Adiposity Diseases, Department of Psychosomatic Medicine and Psychotherapy, University of Leipzig Medical Center, Leipzig, Germany; ^3^Department of Psychosomatic Medicine, University Medical Center of Johannes Gutenberg University Mainz, Mainz, Germany; ^4^Department of Applied Human Sciences, Magdeburg-Stendal University of Applied Sciences, Stendal, Sachsen-Anhalt, Germany

**Keywords:** fatigue, multidimensional fatigue inventory, MFI-20, validation, German norm values

## Abstract

**Objective:**

The Multidimensional Fatigue Inventory (MFI-20) is commonly used, but its factor structure remains unclear. The MFI-20 consists of five subscales (general fatigue, physical fatigue, reduced activity, reduced motivation, and mental fatigue). This study investigates the psychometric properties, including the factor structure, of a general German population sample and tests group hypotheses on gender and age. Another objective is to provide normative data by gender and age groups.

**Methods:**

Using data from a representative German sample (n=2,509), reliability and convergent validity measures, group hypothesis testing, and confirmatory/exploratory factor analyses were conducted.

**Results:**

The MFI-20 demonstrated satisfactory internal consistency and showed adequate convergent validity with the SF-36. All subscales of the MFI-20 were significantly correlated (0.71–0.85). Physical fatigue exhibited the highest (0.42) and mental fatigue had the lowest (0.19) correlation with age. Fatigue scores were significantly higher for women and significantly increased with age. A five-factor structure showed poor model fit; using an exploratory factor analysis, a two-factor structure emerged (a general factor and a mental/motivational factor).

**Conclusion:**

The MFI-20 is a reliable and valid instrument for measuring fatigue in the general population, but the five-factor structure is not supported. The subscale general fatigue or the MFI-20 total score might measure fatigue sufficiently. The provided norms can be used for further research and individual assessment.

## Introduction

Definitions of fatigue often include “general exhaustion, extreme tiredness, weakness, and lack of energy” (1) and “impaired physical and/or cognitive functioning” (2). It is both a normal, transient phenomenon in the general population and one of the most-stated symptoms in (chronic) pathological conditions. Chronic fatigue, as an enduring state with pathological significance that does not reduce through common mechanisms of regeneration, is stated as one of the most frequent symptoms in cancer, lupus, chronic inflammation, and patients with multiple sclerosis (3, 4). It is also a prominent symptom in psychiatric disorders, especially in major depression (5, 6). Recently, fatigue has been described as part of the post-COVID-19 syndrome (7). Interestingly, research on myalgic encephalomyelitis/chronic fatigue syndrome (ME/CFS) shows heterogeneous but converging evidence for a “neuropsychological profile” (8), i.e., substantial differences to healthy controls using different neuroimaging techniques (9). Fatigue is different from fatigability, an objective change in performance that can be measured electrophysiologically but is not necessarily correlated with the subjective experience of fatigue (10). Objective somatic markers or correlates of fatigue have yet to be found (2) which shows the importance of valid and reliable self-report instruments, e.g., patient-reported outcome measures (PROMs). There is an ongoing discussion on the dimensionality of fatigue. It is often described as two-dimensional (mental and physical) or three-dimensional (physical, emotional, and cognitive) (11). More than 40 fatigue measurements are in general use (12). Instruments to directly compare the results of different fatigue instruments have been developed (13). Alongside unidimensional fatigue inventories, the most studied multidimensional fatigue inventories are the Checklist Individual Strength, the Chalder Fatigue Scale, the Multidimensional Assessment of Fatigue, the Piper Fatigue Scale, and the Multidimensional Fatigue Inventory (*MFI-20*). The latter is the most used of those consisting of three or more subscales (14) and is further investigated in this study.

The *MFI-20* was originally developed focusing on patients with cancer and initially proposed a five-factor structure, namely, general fatigue, physical fatigue, reduced activity, reduced motivation, and mental fatigue (15). This structure is strongly questioned by many studies, finding a three-, four- or five-factor structure with varying item loadings [refer to (11) for an overview]. A recent German study of patients (*n* = 140) with spinal muscular atrophy, however, confirmed the five-factor structure using principal component analysis (16). They explained 62.2% of the variance, and only five of the 20 items did not load on the components that otherwise matched the original *MFI-20* subscales. Contrary to validation studies of the *MFI-20* in disease-specific populations, reliability and validity of the *MFI-20* in large general population samples are scarce. Large German, US-American, and Dutch studies show subscale-specific age and gender differences (1, 11, 17). The last German validation study was published in 2003 and relied on data from 1998. Hence, updated and more detailed results are warranted for research and practice. This study uses a large representative German population sample to (a) investigate the psychometric properties of the *MFI-20*, including the factor structure, (b) test group differences between age and gender, and (c) provide German population-based norms.

## Methods

### Sampling

A random sample of German residents aged 16 and older was recruited as part of a broader cross-sectional questionnaire survey between October and December 2021. Since there is no directory available that contains the addresses of all private households or individuals in Germany, the “ADM Sampling System for face-to-face surveys” (18) was used to draw a representative German sample. A market and social research company (USUMA GmbH, Berlin, Germany) performed the subject acquisition and face-to-face interviews with trained interviewers. Overall, 5,901 persons were contacted, 2,526 participated (others did not respond or were ill or otherwise unavailable at the appointed interview date; all reasons for non-participation are listed at the end of the manuscript), and 2,509 completed the interview and the self-report battery. Data for comparison on demographics between participants and non-participants were not available, but the participants are representative regarding gender, age, and regional distribution (16 German federal states). All data were fully anonymized by USUMA prior to data analysis. All participants gave their verbal informed consent in accordance with the Helsinki declaration, which was documented by USUMA, and followed a structured face-to-face interview to collect the statistical and sociodemographic data. Participants were ≥ 16 years and are legally competent in Germany to participate in a study without the need for others' consent. The self-report questionnaires, such as the *MFI-20*, were then completed *via* paper and pencil independently by the subjects themselves, so as to not bias their personal mental and physical health answers. The study was approved by the Ethical Review Committee of the University of Leipzig (298/21-ek).

### Measurements

The Multidimensional Fatigue Inventory (*MFI-20*) is a 20-item self-report instrument, which consists of five subscales: 1. *general fatigue* (items 1, 5, 12, 16), 2. *physical fatigue* (items 2, 8, 14, 20), 3. *reduced activity* (items 3, 6, 10, 17), 4. *reduced motivation* (items 4, 9, 15, 18), and 5. *mental fatigue* (items 7, 11, 13, 19). Each subscale consists of four items with possible answers on a 5-point Likert scale (1 = yes, that is true; 5 = no, this is not true). Higher scores indicate higher levels of fatigue. Convergent validity with a Visual Analog Scale (VAS) assessing fatigue showed significant correlations ranging from 0.77 (general fatigue) to 0.23 (mental fatigue) (15), with the Fatigue Severity Scale ranging from 0.73 (general fatigue) to 0.16 (reduced activity) (19). The previous German validation study argued for using the total score of the 20 items because of the empirically uncertain factorial structure and the strong correlations between the subscales (1).

The Short Form-36 Health Survey (*SF-36*) (20) was used to provide a convergent validity criterion for the *MFI-20*. The 36 questions on the *SF-36* are meant to reflect eight domains of health, including physical functioning, physical role, pain, general health, vitality, social functioning, emotional role, and mental health (range 0 to 100). Additionally, a Physical Composite Score (PCS) and a Mental Composite Score (MCS) can be calculated. These scores are T-values (mean = 50, SD = 10) in reference to a US norm sample. Especially, the *SF-36* domain *vitality* has been widely used as a fatigue marker (21, 22).

### Statistical analysis

IBM SPSS Statistics version 28 (23) was used for the statistical analyses. Negatively phrased items were inverted prior to further analyses. Because the questionnaire scores were not normally distributed (Shapiro-Wilk tests for all fatigue scales, *p* < 0.001), median and mode values are reported in addition to means and standard deviations (SD). Spearman's ρ correlations were calculated to investigate the association among the *MFI-20* subscales and between those, the *SF-36* domains, and age. Mann–Whitney *U* tests were calculated to compare *MFI-20* scores between male and female subjects. Kruskal-Wallis tests with Dunn-Bonferroni *post-hoc* tests were conducted to compare seven age groups (≤ 24, 25–34, 35–44, 45–54, 55–64, 65–74, and ≥ 75 years, in accordance with other studies using this survey). A confirmatory factor analysis was computed using the lavaan R package for SPSS (24). The norm tables were conducted as percentile ranks. McDonald's omega (ω_t_), an alternative measure of internal consistency that does not assume a tau-equivalent but a congeneric model, was computed using the SPSS Macro OMEGA (25).

## Results

### Participants' sociodemographic and psychometric data

Table 1 summarizes the sociodemographic characteristics, shows the *MFI-20* subscale and total scores, and the *SF-36* scores of the total sample. The descending order of the *MFI-20* subscales, with general fatigue exhibiting the highest scores and mental fatigue the lowest scores, is comparable to a recent Dutch norm sample and other studies (11). The five *MFI-20* subscales, the total score, and age did not follow a normal distribution. The *MFI-20* total score had a profound right-tailed skewness (0.82) and a slight flat kurtosis (−0.13). The five subscales showed a strong floor effect, i.e., between 15% (e.g., general fatigue) and 20% (e.g., mental fatigue) of the subjects exhibited the lowest possible score.

**Table 1:** Sociodemographic characteristics, MFI-20 and SF-36 scores for the total sample (*N* = 2,509), and for the male participant and female participant.

	**Total *N* = 2,509**	**Male *n* = 1,230 (49.0%)**	**Female *n* = 1,276 (50.9%)**
Age range 16–95; M (SD)	49.48 (17.81)	49.85 (17.77)	49.16 (17.83)
Marital status *n* (%)			
Married	1,165 (46.4)	597 (48.6)	557 (44.4)
Not married	1,340 (53.4)	631 (51.4)	707 (55.4)
Education *n* (%)			
No graduation	52 (2.1)	20 (1.6)	32 (2.5)
Primary education	1,729 (68.9)	845 (68.7)	881 (69.0)
High school and vocational education	451 (18.0)	229 (18.6)	222 (17.4)
College / university	231 (9.2)	114 (9.3)	117 (9.2)
Currently student	46 (1.8)	22 (1.8)	24 (1.9)
Employment *n* (%)			
Full-time	1,150 (45.8)	693 (56.3)	455 (35.7)
Part-time	310 (12.4)	46 (3.7)	264 (20.7)
Other	77 (3.1)	15 (1.2)	61 (4.8)
In vocational education or training	156 (6.2)	79 (6.4)	77 (6.0)
Unemployed	189 (7.5)	76 (6.2)	113 (8.9)
Retired	622 (24.8)	320 (26.0)	302 (23.7)
*MFI-20* scores M (SD)			
General fatigue	8.66 (3.92)	8.20 (3.77)	9.08 (4.00)
Physical fatigue	8.45 (4.28)	8.11 (4.23)	8.77 (4.30)
Reduced activity	8.36 (4.00)	8.19 (3.97)	8.51 (4.04)
Reduced motivation	8.13 (3.48)	7.99 (3.41)	8.26 (3.55)
Mental fatigue	8.08 (3.49)	7.83 (3.48)	8.32 (3.48)
Total score	41.69 (17.54)	40.36 (17.30)	42.98 (17.65)
*SF-36* scores M (SD)			
Physical functioning	89.27 (19.98)	90.40 (18.88)	88.17 (20.95)
Physical role	83.81 (31.99)	85.44 (30.68)	82.25 (33.13)
Pain	82.00 (24.60)	84.03 (23.36)	80.06 (25.57)
General health	69.66 (22.46)	71.20 (21.96)	68.16 (22.85)
Vitality	66.60 (21.00)	69.01 (20.14)	64.30 (21.52)
Social functioning	88.28 (20.20)	89.98 (18.57)	86.68 (21.44)
Emotional role	88.04 (28.67)	91.26 (24.29)	84.96 (32.04)
Mental health	77.04 (17.85)	79.39 (16.52)	74.80 (18.74)
Physical composite score (PCS)	51.09 (9.91)	51.49 (9.68)	50.69 (10.13)
Mental composite score (MCS)	51.85 (9.37)	53.09 (8.22)	50.66 (10.19)

Note that the reported percentages refer to valid cases. M, mean; SD, standard deviation. Note that n = 3 identified as divers.

### Item analysis

Item scores ranged from 1.75 (item 18) to 2.38 (item 20). The selectivity (item-scale correlations) varied between 0.545 (item 9) and 0.814 (item 14), and the homogeneity (inter-item-correlations) varied between 0.362 (item 9 x item 15) and 0.733 (item 8 x item 20). The mean value of missing answers per item was 6 (SD = 2.77) or 0.24%, indicating a highly accepted questionnaire. Item characteristics are shown in Table 2.

**Table 2:** Item characteristics of the multidimensional fatigue inventory (MFI-20).

**Subscale**	**Item**	**M**	**SD**	**Skewness**	**kurtosis**	**% m**.	**r_it_**	**α if deleted**
General fatigue	1	2.03	1.18	0.97	−0.11	0.36	0.79	0.961
	12	2.31	1.07	0.59	−0.33	0.08	0.69	0.962
	5	2.21	1.17	0.74	−0.41	0.12	0.74	0.962
	16	2.11	1.18	0.83	−0.35	0.08	0.80	0.961
Physical fatigue	8	2.17	1.17	0.84	−0.21	0.20	0.79	0.961
	20	2.38	1.27	0.65	−0.64	0.16	0.76	0.961
	2	1.99	1.23	1.05	−0.08	0.32	0.75	0.961
	14	1.92	1.21	1.13	0.11	0.36	0.82	0.961
Reduced activity	3	2.23	1.16	0.71	−0.37	0.20	0.82	0.961
	6	2.21	1.14	0.75	−0.25	0.16	0.76	0.961
	10	1.95	1.17	1.06	0.11	0.44	0.74	0.961
	17	1.98	1.19	1.03	−0.03	0.32	0.83	0.960
Reduced motivation	4	2.07	1.10	0.83	−0.12	0.28	0.72	0.962
	15	2.36	1.14	0.52	−0.55	0.16	0.64	0.963
	9	1.97	1.13	1.04	0.17	0.36	0.63	0.963
	18	1.75	1.07	1.32	0.78	0.44	0.73	0.962
Mental fatigue	7	1.92	0.98	1.04	0.64	0.20	0.75	0.962
	11	1.97	0.99	0.88	0.18	0.12	0.74	0.962
	13	2.02	1.15	1.00	0.03	0.20	0.66	0.962
	19	2.18	1.14	0.70	−0.43	0.28	0.61	0.963

M, mean; SD, standard deviation; % m., % missing; r_it_, corrected item-total correlation; α if deleted, Cronbach's α if the item is deleted.

### Reliability

Ascertaining reliability, Cronbach's α values for the subscales were 0.87 (General fatigue), 0.90 (Physical fatigue), 0.88 (Reduced activity), 0.79 (Reduced motivation), and 0.83 (Mental fatigue). These values are similar to the developers' 1995 values (15) and follow the identical ordinal ranking found in the first German 2003 validation study (1). The omission of items did not increase any Cronbach's α considerably. McDonald's ω_t_ for all items was 0.964, indicating high reliability (26, 27).

### Convergent validity

The convergent validity of the *MFI-20* was examined by correlating the *MFI-20* subscales/total score with the *SF-36* domains and its PCS and MCS. All correlations between the *MFI-20* subscales/total score and the *SF-36* domains/PCS/MCS were significant (*p* < 0.001), using Spearman's ρ correlations due to the non-parametric distribution. Correlations between 0.40 and 0.60 can be seen as a medium in convergent validity. As expected, vitality exhibited the highest correlations with three of the five *MFI-20* subscales and the total score, highlighted in bold in Table 3. The PCS correlated the most with physical fatigue (−0.72) and the lowest with mental fatigue (−0.41). The MCS correlated the most with the *MFI-20* total score (−0.64), followed by general fatigue (−0.63) and mental fatigue (−0.61). Pairwise correlations of the *MFI-20* subscales ranged between 0.71 and 0.85 and are slightly lower when controlled for age (refer to Table 3, values labeled ^p^). The correlation between the *MFI-20* total score and age was r_s_ = 0.35, with physical fatigue having the highest (r_s_ = 0.42) and mental fatigue having the lowest (r_s_ = 0.19) subscale correlation with age (all *p* < 0.001).

**Table 3:** Correlations between the MFI-20 scores, age, and SF-36 domains/composite scores.

	**General fatigue**	**Physical fatigue**	**Reduced activity**	**Reduced motivation**	**Mental fatigue**	**Total score**
*MFI-20*						
General fatigue	-	0.84	0.82	0.78	0.76	0.92
Physical fatigue	0.83^p^	-	0.85	0.77	0.71	0.92
Reduced activity	0.80^p^	0.83^p^	-	0.83	0.79	0.94
Reduced motivation	0.76^p^	0.75^p^	0.81^p^	-	0.78	0.91
Mental fatigue	0.75^p^	0.71^p^	0.78^p^	0.78^p^	-	0.88
Total score	0.91^p^	0.91^p^	0.93^p^	0.90^p^	0.88^p^	-
**Age**	0.32	0.42	0.33	0.32	0.19	0.35
***SF-36***						
Physical functioning	−0.55	−0.66	−0.56	−0.49	−0.42	−0.59
Physical role	−0.58	−0.65	−0.57	−0.50	−0.44	−0.60
Pain	−0.62	−0.70	−0.58	−0.51	−0.45	−0.64
General health	−0.70	**−0.76**	−0.67	−0.63	−0.55	−0.73
Vitality	**−0.78**	−0.73	**−0.69**	**−0.67**	−0.62	**−0.76**
Social functioning	−0.61	−0.62	−0.59	−0.55	−0.54	−0.64
Emotional role	−0.48	−0.44	−0.45	−0.43	−0.44	−0.49
Mental health	−0.69	−0.63	−0.65	−0.65	**−0.66**	−0.72
PCS	−0.59	−0.72	−0.58	−0.50	−0.41	−0.62
MCS	−0.63	−0.53	−0.57	−0.58	−0.61	−0.64

Upper right values show Pearson's correlations. Lower left values (^p^) show partial correlations (controlled for age).

All correlations are significant (p < 0.001), using Spearman's ρ, also significant results with Kendall's τ and Pearson's correlations. The highest correlation between the MFI-20 scores and the SF-36 domains are printed in bold. SF-36, Short Form (36) Health Survey; PCS, Physical Component Scale; MCS, Mental Component Scale.

### Comparisons by gender and age groups

Mann–Whitney *U* tests showed significantly higher fatigue scores in females for all but one subscale and the total score (all *p* < 0.001, reduced activity *p* = 0.04) but not for reduced motivation (*p* = 0.08). The Kruskal-Wallis test showed significant differences between age groups for the different subscales and the total score: general fatigue [*H*(2) = 269.969], physical fatigue [*H*(2) = 466.147], reduced activity [*H*(2) = 318.654], reduced motivation [*H*(2) = 273.402], mental fatigue [*H*(2) = 118.925], and the total score [*H*(2) = 342.471]; all *p* < 0.001, *df* = 6. Following *post-hoc* tests (Dunn-Bonferroni) revealed which age groups significantly differed from each other (Table 4).

**Table 4:** Results of the Kruskal-Wallis Dunn-Bonferroni *post-hoc* tests for the MFI-20 subscales and the total score, by age groups, shown in the superscript table.

***MFI-20* subscale / age groups [years]**	**≤24**	**25–34**	**35–44**	**45–54**	**55–64**	**65–74**	**≥75**
General fatigue	7.07^a^	7.34^a^	8.25^b^	8.20^b^	9.21^c^	9.54^c^	11.70^d^
Physical fatigue	6.39^a^	6.58^a^	7.67^b^	7.72^b^	9.05^c^	9.95^d^	13.16^e^
Reduced activity	7.00^a^	7.01^ab^	7.72^b^	7.65^b^	8.69^c^	9.56^d^	12.20^e^
Reduced motivation	6.83^a^	6.92^a^	7.76^b^	7.73^b^	8.55^c^	8.99^c^	10.85^d^
Mental fatigue	7.52^a^	7.28^ab^	7.95^ab^	7.67^bc^	8.28^bc^	8.51^c^	10.05^d^
*MFI-20* Total score	34.80^a^	35.11^a^	39.35^b^	39.00^b^	43.83^c^	46.54^c^	57.97^d^

Note that non-differing age groups denote the same letters/colors; hence significantly differing age groups denote different letters/colors. Overlaps can occur due to the pairwise *post-hoc* tests.

**Figure 1:**
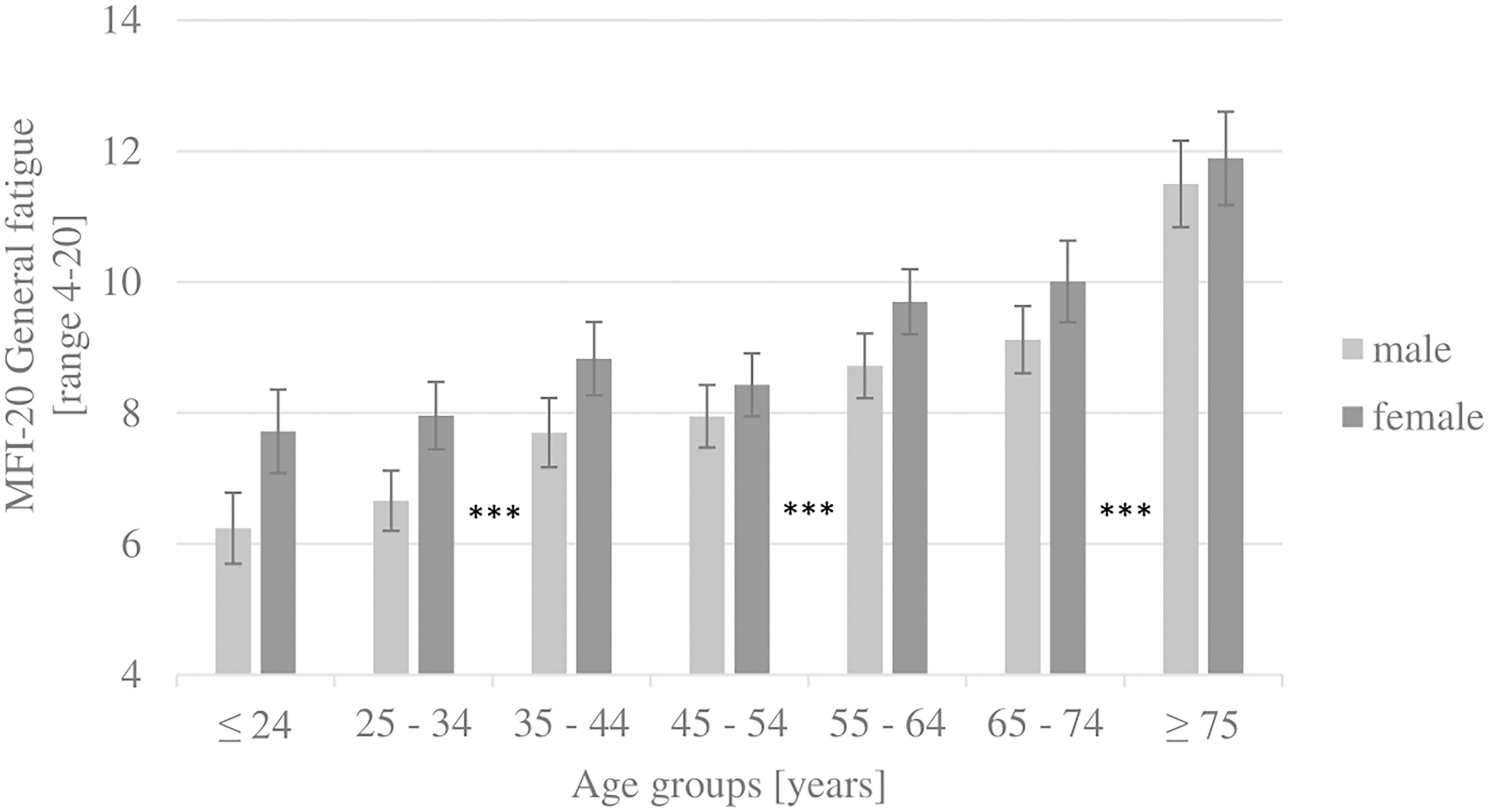
Results of the subscale general fatigue, by gender and age groups (whiskers show 95% confidence intervals). ***post-hoc tests (Dunn-Bonferroni): *p* < 0.001.

**Figure 2:**
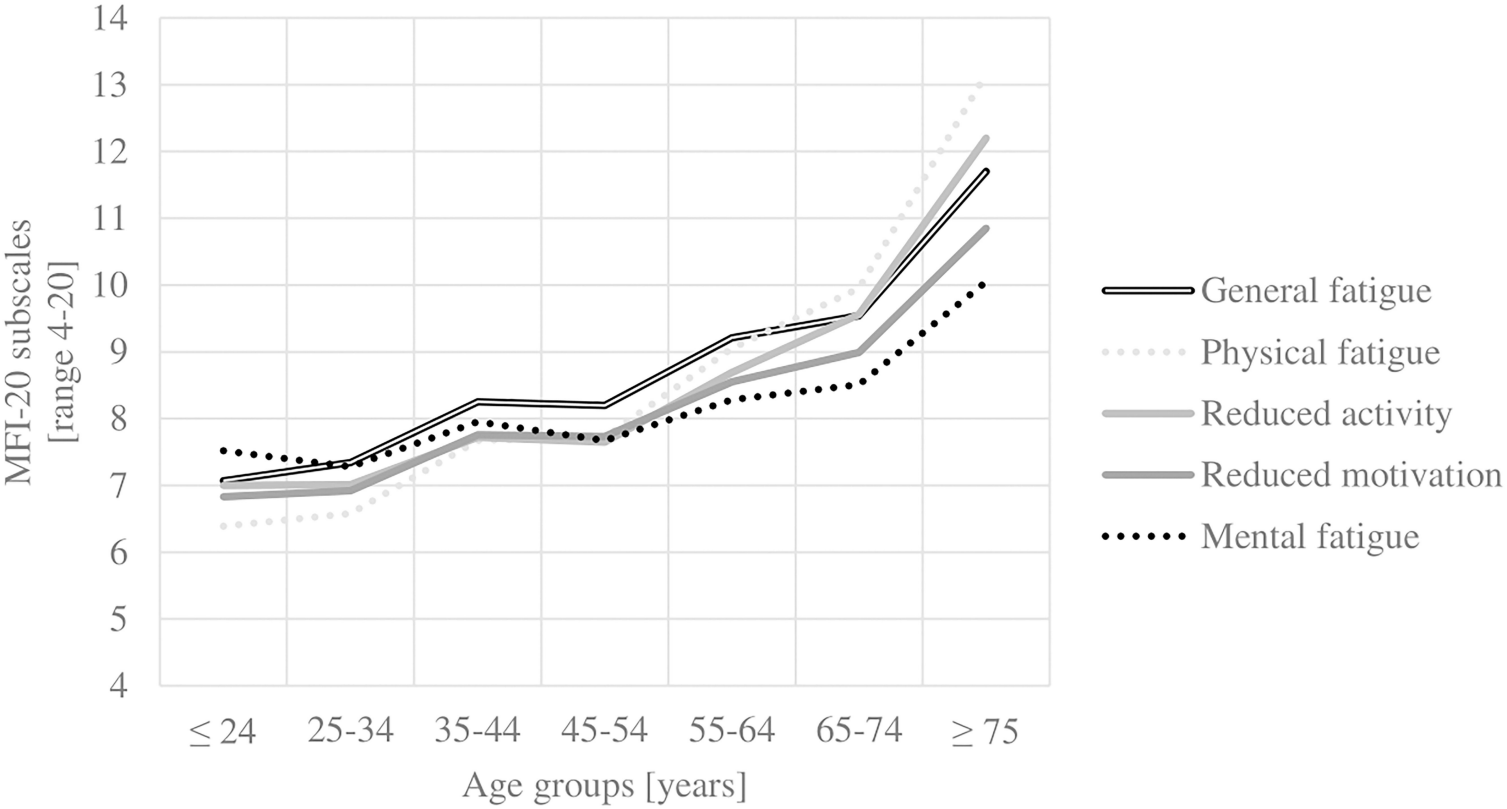
MFI-20 subscales over the course of age.

Two-way ANOVAs showed no interaction effect between age (seven age groups) and gender (male and female) on any subscale and the total score. However, this must be interpreted carefully due to non-parametric distributions. A descriptive, insignificant interaction effect of gender and age groups 3–44 and 45–54 years could be observed on all subscales (except mental fatigue) and the total score. Between these two age groups, the scores increase with age for men but decrease with age for women. Figure 1 shows the general fatigue scores to highlight the age and gender structure of the results. Figure 2 illustrates that in the oldest age group, reduced motivation and mental fatigue, two cognitive appraisal domains, show the relatively lowest scores. In the youngest age group (≤ 24 years), mental fatigue exhibited the highest and physical fatigue the lowest scores. In contrast, in the oldest age group (≥ 75 years), mental fatigue exhibited the lowest and physical fatigue the highest scores.

### Factor analyses, construct validity

The data did not follow a multivariate normal distribution (28). A confirmatory factor analysis (CFA) for the five-factor structure showed poor model fit, comparative fit index (CFI) = 0.891, Tucker-Lewis index (TLI) = 0.871 (values > 0.95 indicate an acceptable fit), and root mean square error of approximation (RMSEA) = 0.105 (values < 0.08 indicate an acceptable fit). The Kaiser-Meyer-Olkin criterion of sampling adequacy was highly developed (0.972). The Bartlett test on sphericity was significant (*p* < 0.001, approximate χ^2^ = 39625.349, *df* = 190). An exploratory factor analysis (EFA using principal component analysis) showed two disparate components with an eigenvalue (λ) > 1. The first component with λ = 11.872 explained 59.36% of the variance, and the second component with λ = 1.302 explained 6.51% of the variance. Another EFA, using varimax rotation, a factor loading > 0.70, and a cross-loading of < 0.40, led to the following solution: a general factor with items from all five subscales (items 3, 8, 20, 4, 1, and 15 in descending loading order, all items phrased positively) and a mental/motivational factor with items from the subscales mental fatigue and reduced motivation (items 13, 9, 18, and 19 in descending order, all items phrased negatively). Additionally, the goodness-of-fit test for a unifactorial solution, computed with Hayes' macro (25) with all items, was significant (*p* < 0.001, χ^2^ = 5457.688, *df* = 170).

### Norm tables

Given the significant differences between the *MFI-20* subscales across gender and age, we calculated percentile ranks based on the *MFI-20* subscale raw scores for gender and age separately. The percent rank norms as a non-linear and distribution-free test value transformation were applied since the distributions displayed a profound right-tailed skewness. A percent rank PR_n_ shows the percentage of the norm sample that scored lower and exactly as the subject n, i.e., $PR_{n}=100^{*}\frac{freq_{cum}\left(x_{n}\right)}{N}$. The *MFI-20* norm tables are shown in the Supplementary material for the subscales and the total score, separately by gender and age group.

## Discussion

The *MFI-20* shows satisfying psychometric properties, including reliability and convergent validity. The construct validity was examined using CFA, which could not replicate the five-factor structure. An EFA suggests a strong *general fatigue* factor and a smaller *mental/motivational* factor. Kieffer et al. found a four-factor structure but allowed significant cross-loadings, “indicating that these items are unstable” (11). It has been shown that shorter versions, e.g., an MFI-10, resulted in comparable or better fit indices (29, 30). Regarding the reported factor structure, the above-mentioned clustering of positive and negative items was also noted by others (11, 30). The *MFI-20* scores are overall comparable with other studies, showing higher fatigue scores for females and increasing scores with age. In comparison with a recent Dutch norm sample (11), the German scores are lower in all five subscales; most pronounced differences, mean (SD), were found for reduced activity, 9.3 (3.9) vs. 8.36 (4.00), and the total score 44.9 (16.7) vs. 41.69 (17.54). These differences may show overall lower fatigue values in the German population, but effects of language (item formulation), differences in introspection, acceptance of one's own experiences, social desirability, and/or the date of data acquisition (refer to limitations) may have contributed to these differences.

Reduced motivation showed the lowest internal consistency, which is in line with many studies (16). Mental fatigue showed the lowest interscale correlations with the other subscales. The highest interscale correlations were found between physical fatigue and general fatigue, and physical fatigue and reduced activity, exactly as in other studies (1, 31). This is in line with reported difficulties to distinguish between general and physical fatigue (16) and argues for combining these two subscales or even for using the entire unidimensional structure of the *MFI-20*. Since even the lowest subscale × total score correlation is higher than any inter-subscale correlation in our data, it could be that “the MFI-total is a more valid score for fatigue than the single MFI-subscale scores” (31).

The right-tailed skewness of the distribution in the norm sample may be less pronounced if more items with varying difficulties per subscale existed. The observed floor effect of 15–20% is similar to a comparable general sample: Lin et al. (17) compared subsamples with chronic fatigue symptoms, who had moderate ceiling percentages (1.4–13.0%) and negligible floor percentages (0–3.8%) in the subscales, with a (not further defined) “well” subsample from the general population. They in turn showed negligible ceiling percentages (0–0.5%) but substantial floor percentages (10.3–26.5%), lying in a slightly broader but comparable range with our data. Varying floor/ceiling effects in fatigue norm samples have been observed (32), which could be of diagnostic interest in different pathological subgroups. In a sample of cancer patients receiving radiotherapy, 28.6% showed a ceiling effect in general fatigue and 17.3% showed a floor effect in mental fatigue (33). In a sample of patients with spinal muscular atrophy, 19.3% exhibited a floor effect in mental fatigue (16). These strong differences in percentages of floor and ceiling effects between the subscales, even within samples, question the homogeneity of the latent construct underlying the *MFI-20*. The lowest floor effect in the cited healthy US-American sample (17) was in the subscale of general fatigue, indicating the best diagnostically conclusive discrimination of this subscale. This supports arguments stating that the subscale general fatigue measures fatigue sufficiently valid and that the other subscales might measure neighboring constructs, such as physical or cognitive functioning (11). In comparison to other fatigue inventories, the items of the *MFI-20* focus on cognitive appraisal rather than objectifiable fatigue impacts, and they do not express an explicit attribution of the stated experiences of fatigue. This is supported by the Mental Composite Score (MCS) of the *SF-36*, which correlates most strongly with the *MFI-20* total score, followed by the general fatigue and mental fatigue subscales. Furthermore, general fatigue correlated higher with the MCS than with the PCS.

The strength of our study is the use of a large German population-based sample, which was representative regarding gender, age, and regional distribution. Representativeness regarding education level, employment, and marital status was not considered. Further study limitations include the lack of a systematic comparison between chronically ill people and the general population. For example, the age effect of fatigue in the general population could not be found in cancer patient samples (34), for whom the *MFI-20* was originally developed. Data on somatic symptoms or psychopathology would have been interesting but are limited in validity as part of a self-report battery. A simultaneous standardization process for the general population and selected patient groups would provide suggestions as to whether different populations could profit from adapted versions of the *MFI-20*. Furthermore, more indices for determining convergent validity would have been preferable. Another limitation might be the acquisition of subjects during the COVID-19 pandemic. There could be a selection bias, as more vulnerable, more cautious, and potentially more fatigued subjects might have refused to participate in the study. This potential exclusion may partially explain the observed overall lower *MFI-20* scores in comparison with other studies (11), where data acquisition occurred before the pandemic. One potential explanation is that the pandemic did not increase fatigue in the general population; another explanation is that the pandemic did indeed increase fatigue, but the aforementioned exclusion bias overshadowed this effect. Since the data are cross-sectional, no statements on sensitivity to (clinical) changes in fatigue can be made. The developers of the *MFI-20*, however, state that for healthy subjects, the subscale general fatigue is the most sensitive to change and, thus, state that “it could be argued that when a short instrument is required only this scale should be used” (15). Once again, this supports the assumption that the subscale general fatigue might measure fatigue sufficiently enough.

### Reasons for non-participation of subjects

The kish selection grid was used to identify randomly assigned target persons. A total of 57.2% of the target population did not participate due to the following reasons: household did not respond within four attempts (*n* = 791, 13.4%); household refused participation (*n* = 1374, 23.3%); target person did not respond within four attempts (*n* = 288, 4.9%); target person was on a business or vacation trip (*n* = 61, 1.0%); target person was ill (*n* = 75, 1.3%); and target person refused participation (*n* = 786, 13.3%).

## Data availability statement

The original contributions presented in the study are publicly available. This data can be found here: https://doi.org/10.26068/mhhrpm/20221017-000.

## Ethics statement

The studies involving human participants were reviewed and approved by Ethical Review Committee of the University of Leipzig (298/21-ek). Written informed consent for participation was not required for this study in accordance with the national legislation and the institutional requirements.

## Author contributions

Conceived and designed the data acquisition: EB. Analyzed the data: AW and MZ. Contributed reagents, materials, and analysis tools: EB, MM, and MZ. Wrote the manuscript: AW. Interpretation of the data: AW, MN, MM, EB, and MZ. All authors contributed to the manuscript revision, read, and approved the submitted version.
