# Supplementary material for: Psychometric properties, factor structure, and German population norms of the multidimensional fatigue inventory (MFI-20)
Source: Front Psychiatry. 2022 Dec 20;13:1062426. doi: 10.3389/fpsyt.2022.1062426 (PMC9807811; doi:10.3389/fpsyt.2022.1062426)
Supplement: Supplementary file 1 [file Data_Sheet_1.PDF]

Supplementary material norm tables

Table 1. Percent ranks of the MFI-20 subscales, by gender. 2
Table 2. Gender-specific percent rank norms for all n ≤ 24 years. 3
Table 3. Gender-specific percent rank norms for all n 25-34 years. 4
Table 4. Gender-specific percent rank norms for all n 35-44 years. 5
Table 5. Gender-specific percent rank norms for all n 45-54 years. 6
Table 6. Gender-specific percent rank norms for all n 55-64 years. 7
Table 7. Gender-specific percent rank norms for all n 65-74 years. 8
Table 8. Gender-specific percent rank norms for all n ≥ 75 years. 9
Table 9. Percent ranks of the MFI-20 sum score, by gender and age groups. 10

Annotations

The norm tables are colored as followed:
Darkest gray: First value ≥ 50, approximately marking the mean value
Middle gray: Values within the first SD (15.9 – 84.1 % of the distribution)
Lightest gray: Values within the second SD (2.4 – 97.6 % of the distribution)

Abbreviations

M: mean, SD: standard deviation, mdn: median
GF: General fatigue
PF: Physical fatigue
RA: Reduced activity
RM: Reduced motivation
MF: Mental fatigue

Table 1. Percent ranks of the MFI-20 subscales, by gender.

	General fatigue			Physical fatigue			Reduced activity			Reduced motivation			Mental fatigue		
Sub-scale score	Total n=2506 3 m.	Male n=1230 0 m.	Female n=1273 3 m.	Total n=2505 4 m.	Male n=1228 2 m.	Female n=1274 2 m.	Total n=2502 7 m.	Male n=1225 5 m.	Female n=1274 2 m.	Total n=2504 5 m.	Male n=1228 2 m.	Female n=1273 3 m.	Total n=2505 4 m.	Male n=1229 1 m.	Female n=1273 3 m.
4	15	18	13	20	23	17	18	20	17	15	15	14	20	23	17
5	26	31	22	33	37	29	31	32	29	28	28	27	28	32	25
6	37	42	32	44	49	39	42	44	41	40	40	40	38	42	35
7	46	51	42	52	56	49	52	53	50	51	53	50	49	52	45
8	57	62	53	61	65	58	60	62	59	61	64	58	62	65	59
9	64	68	60	67	70	63	67	68	66	69	71	67	70	72	67
10	71	75	67	72	75	69	72	74	70	76	78	74	76	78	74
11	76	80	72	76	79	74	78	79	77	82	83	80	82	84	80
12	81	85	78	81	83	79	83	84	83	88	89	86	87	88	87
13	86	88	83	84	86	83	87	88	86	92	93	90	92	92	91
14	90	92	87	88	88	87	91	91	90	94	95	94	95	95	94
15	93	95	91	91	91	90	93	93	92	96	96	96	96	97	96
16	96	97	95	94	94	93	95	96	95	98	98	98	98	98	98
17	98	98	97	96	96	96	97	97	96	99	98	99	99	99	99
18	99	99	99	97	98	97	98	98	98	99	99	100	99	100	99
19	99	99	100	99	99	99	99	99	99	100	100	100	100	100	100
20	100	100	100	100	100	100	100	100	100	100	100	100	100	100	100
M	8.66	8.20	9.09	8.45	8.11	8.78	8.36	8.21	8.51	8.13	7.99	8.27	8.08	7.83	8.32
SD	3.92	3.77	4.00	4.28	4.23	4.30	4.00	3.96	4.04	3.48	3.48	3.54	3.49	3.48	3.48
Med	8	7	8	7	7	8	7	7	7	7	7	8	8	7	8
Mod	4	4	4	4	4	4	4	4	4	4	4	4	4	4	4
Note. For each column the missing values (m.) are shown.															

Table 2. Gender-specific percent rank norms for all $n \leq 24$ *years*.

	Male					Female				
Raw score	GF	PF	RA	RM	MF	GF	PF	RA	RM	MF
4	36	47	32	27	27	20	32	22	22	22
5	56	68	44	47	40	37	49	41	41	26
6	67	80	61	62	54	47	60	52	58	40
7	77	81	71	69	66	54	65	63	66	52
8	83	90	80	78	75	66	74	73	75	63
9	87	94	86	81	80	73	78	81	78	73
10	90	94	88	89	83	81	83	83	84	79
11	93	95	91	94	89	86	88	86	92	87
12	95	97	93	96	93	88	93	91	96	93
13	98	97	97	97	95	92	94	93	98	95
14	98	98	99	97	96	94	96	95	98	96
15	98	98	99	98	96	97	98	98	98	98
16	98	98	99	98	98	98	98	99	99	98
17	100	98	99	99	99	98	98	99	100	98
18	100	98	99	99	100	99	99	99	100	98
19	100	99	100	100	100	100	99	100	100	99
20	100	100	100	100	100	100	100	100	100	100
M	6.24	5.67	6.61	6.68	7.09	7.72	6.97	7.27	6.94	7.83
SD	2.84	2.79	2.97	2.97	3.25	3.54	3.46	3.33	2.89	3.31
mdn	5	6	6	6	6	7	6	6	6	7
mode	4	4	4	4	4	4	4	4	4	4
Note. M: mean, SD: standard deviation, mdn: median. Male n=108. Female n=120.										

Table 3. Gender-specific percent rank norms for all *n 25-34 years*.

	Male					Female				
Raw score	GF	PF	RA	RM	MF	GF	PF	RA	RM	MF
4	31	45	36	31	31	19	27	24	21	21
5	51	60	52	45	41	30	44	39	39	34
6	63	71	59	59	54	45	57	51	56	42
7	70	76	67	72	66	57	67	64	63	57
8	76	82	76	80	77	66	75	71	72	72
9	81	86	80	87	82	71	80	81	80	78
10	87	92	87	91	89	78	82	83	83	81
11	93	93	91	93	91	83	86	88	87	85
12	95	97	94	96	93	87	91	92	91	90
13	96	97	96	97	94	90	94	94	95	94
14	97	98	98	98	95	92	95	95	95	95
15	98	99	98	99	98	94	97	95	97	97
16	99	100	98	100	98	97	99	96	98	98
17	99	100	99	100	99	99	100	98	99	99
18	99	100	99	100	99	99	100	100	100	99
19	99	100	100	100	99	100	100	100	100	100
20	100	100	100	100	100	100	100	100	100	100
M	6.66	6.03	6.69	6.52	6.92	7.96	7.08	7.31	7.29	7.62
SD	3.14	2.73	3.19	2.73	3.22	3.69	3.38	3.48	3.36	3.40
mdn	5	5	5	6	6	7	6	6	6	7
mode	4	4	4	4	4	4	4	4	4	4

Note. M: mean, SD: standard deviation, mdn: median. Male n=181 (RA n=179). Female n=201 (GF/RM/MF n=200).

Table 4. Gender-specific percent rank norms for all *n 35-44 years*.

	Male					Female				
Raw score	GF	PF	RA	RM	MF	GF	PF	RA	RM	MF
4	22	30	26	16	26	13	22	20	15	19
5	37	45	41	32	35	26	32	34	28	25
6	49	56	52	49	41	37	42	46	40	40
7	57	65	61	62	53	45	56	52	51	48
8	69	73	73	73	65	53	68	63	62	61
9	75	77	78	78	73	60	73	69	72	69
10	82	82	82	82	78	69	77	74	77	74
11	83	84	84	84	85	74	81	82	84	79
12	87	87	88	91	88	81	85	88	90	85
13	90	90	92	92	92	85	89	91	93	91
14	93	91	93	95	95	87	91	92	95	95
15	94	93	94	96	96	91	93	96	97	96
16	96	96	95	98	98	97	97	99	98	99
17	97	97	97	98	99	98	98	99	99	99
18	98	97	97	98	99	99	98	99	99	99
19	99	97	98	99	99	100	99	100	100	100
20	100	100	100	100	100	100	100	100	100	100
M	7.70	7.39	7.47	7.55	7.76	8.83	7.98	7.97	7.99	8.17
SD	3.77	4.01	3.83	3.41	3.53	3.96	3.82	3.64	3.32	3.48
mdn	7	6	6	7	7	8	7	7	7	8
mode	4	4	4	5	4	4	4	4	4	4
Note. M: mean, SD: standard deviation, mdn: median. Male n=198 (PF/RM/MF n=197; RA n=196). Female n=193.										

Table 5. Gender-specific percent rank norms for all *n 45-54 years*.

	Male					Female				
Raw score	GF	PF	RA	RM	MF	GF	PF	RA	RM	MF
4	16	22	21	14	23	14	20	22	15	24
5	28	41	35	29	34	26	37	36	35	34
6	44	54	47	44	46	37	49	49	47	45
7	55	62	57	55	56	50	59	62	59	54
8	67	69	67	67	67	61	65	70	67	66
9	72	74	73	76	76	69	70	74	74	72
10	78	81	79	84	82	74	78	79	78	79
11	83	83	86	88	87	80	83	85	84	83
12	88	85	89	92	90	84	87	90	88	89
13	91	89	90	94	93	87	91	93	93	93
14	93	93	92	95	95	91	93	95	96	94
15	95	95	94	96	97	93	93	96	96	96
16	97	97	98	97	98	94	96	98	98	98
17	98	97	98	98	99	98	98	98	99	99
18	99	98	98	98	99	99	98	98	100	100
19	99	99	98	99	100	99	100	99	100	100
20	100	100	100	100	100	100	100	100	100	100
M	7.95	7.59	7.75	7.74	7.59	8.43	7.83	7.56	7.72	7.75
SD	3.58	3.86	3.72	3.32	3.42	3.82	3.79	3.57	3.42	3.52
mdn	7	6	7	7	7	7	7	7	7	7
mode	4/6	4	4	5	4	4/7	4	4	5	4

Note. M: mean, SD: standard deviation, mdn: median. Male n=218 (PF n=217). Female n=246 (RA n=245).

Table 6. Gender-specific percent rank norms for all *n 55-64 years*.

	Male					Female				
Raw score	GF	PF	RA	RM	MF	GF	PF	RA	RM	MF
4	12	14	14	11	22	7	8	12	12	12
5	24	27	28	22	30	14	19	26	22	19
6	34	41	43	33	38	24	31	38	34	29
7	44	52	52	48	51	33	39	44	44	41
8	59	64	63	58	64	46	51	53	51	58
9	66	70	68	68	73	57	57	63	62	66
10	72	73	72	74	77	61	62	69	70	73
11	78	78	77	79	80	67	68	72	78	80
12	83	81	83	86	84	74	74	78	85	86
13	85	84	87	92	91	81	80	84	89	91
14	90	86	90	94	95	86	85	89	94	96
15	92	89	92	95	96	90	89	92	97	98
16	95	93	94	97	98	95	93	95	98	99
17	97	95	95	98	99	98	96	96	99	100
18	98	97	97	99	100	98	97	98	100	100
19	100	98	100	100	100	99	98	100	100	100
20	100	100	100	100	100	100	100	100	100	100
M	8.72	8.57	8.46	8.46	8.03	9.70	9.52	8.92	8.64	8.52
SD	3.87	4.27	4.05	3.49	3.58	3.92	4.20	4.05	3.48	3.21
mdn	8	7	7	8	7	9	8	8	8	8
mode	8	4	6	7	4	8	6/8	5	4	8

Note. M: mean, SD: standard deviation, mdn: median. Male n=237 (RA n=236). Female n=243 (GF/PF/RM/MF n=242).

Table 7. Gender-specific percent rank norms for all *n 65-74 years*.

	Male					Female				
Raw score	GF	PF	RA	RM	MF	GF	PF	RA	RM	MF
4	12	6	7	7	18	11	7	11	6	11
5	15	15	14	12	24	14	13	16	13	18
6	24	27	29	23	35	19	21	25	25	24
7	35	36	39	38	44	29	30	35	36	31
8	49	49	49	54	61	44	38	44	44	48
9	58	58	57	63	69	49	47	51	58	61
10	67	63	64	71	75	58	58	57	69	74
11	74	70	72	79	82	64	62	66	76	81
12	82	77	77	85	88	72	69	73	84	87
13	89	81	85	92	92	76	74	78	88	92
14	93	86	88	94	96	84	81	86	94	95
15	96	91	92	97	98	89	86	90	96	97
16	97	94	96	99	99	95	88	95	97	98
17	98	96	98	99	99	96	93	96	98	99
18	99	99	99	100	100	98	94	98	100	100
19	100	100	100	100	100	99	97	99	100	100
20	100	100	100	100	100	100	100	100	100	100
M	9.12	9.51	9.33	8.85	8.21	10.01	10.44	9.81	9.15	8.86
SD	3.48	3.87	3.71	3.11	3.29	3.99	4.35	4.04	3.32	3.16
mdn	9	9	9	8	8	10	10	9	9	9
mode	8	8	6	8	4	8	10	4	9	8

Note. M: mean, SD: standard deviation, mdn: median. Male n=180 (RM n=179). Female n=160.

Table 8. Gender-specific percent rank norms for all *n* ≥ *75 years*.

	Male					Female				
Raw score	GF	PF	RA	RM	MF	GF	PF	RA	RM	MF
4	3	2	3	2	13	4	3	3	4	9
5	8	4	4	3	14	6	4	4	7	12
6	9	6	9	8	21	10	6	11	13	16
7	13	9	13	16	28	14	13	18	21	22
8	20	14	16	29	38	21	17	22	27	32
9	27	22	24	37	46	26	22	29	36	37
10	40	32	36	50	57	37	29	34	47	48
11	47	37	41	63	70	41	36	46	54	62
12	56	44	52	74	80	53	39	57	63	71
13	64	51	61	82	88	63	49	62	71	79
14	79	57	75	88	93	73	58	68	80	86
15	93	65	81	93	94	83	63	74	85	89
16	95	77	89	95	98	91	73	77	93	96
17	98	86	91	96	99	93	82	85	96	97
18	99	93	94	99	99	96	86	90	99	98
19	99	95	98	99	99	99	94	95	99	99
20	100	100	100	100	100	100	100	100	100	100
M	11.50	13.06	12.14	10.66	9.62	11.89	13.26	12.27	11.04	10.46
SD	3.46	4.08	3.78	3.20	3.51	3.81	4.36	4.37	3.77	3.74
mdn	12	13	12	10.5	10	12	14	12	11	11
mode	14	16	14	8/10/11	4/11	12	16	11/12	10	11
Note. M: mean, SD: standard deviation, mdn: median. Male n=108. Female n=112.										

Table 9. Percent ranks of the MFI-20 sum score, by gender and age groups.

Sum score	Total	Male Age [yrs]								Female Age [yrs]							
	all	all	≤24	25-34	35-44	45-54	55-64	65-74	≥75	all	≤24	25-34	35-44	45-54	55-64	65-74	≥75
20	5	5	14	11	7	4	1	2	1	4	8	6	5	6	2	1	1
21	8	10	19	22	11	7	3	6	1	6	13	9	6	8	2	3	1
22	10	13	21	28	16	11	6	7	1	8	16	13	9	11	3	4	1
23	13	16	25	31	21	15	9	7	1	10	21	15	11	14	3	4	2
24	16	19	33	35	24	20	12	8	2	13	24	22	13	16	7	8	3
25	20	22	38	41	31	22	13	8	2	17	28	27	18	21	10	11	4
26	22	25	42	44	34	26	18	9	2	20	31	29	22	24	12	13	4
27	25	28	43	46	38	30	20	13	2	23	36	34	27	28	13	13	4
28	29	31	47	51	40	33	24	14	3	26	40	37	33	34	16	13	4
29	31	34	54	55	43	37	27	16	3	29	41	41	34	37	20	15	5
30	34	37	57	58	45	40	30	18	5	32	47	44	37	41	24	17	5
31	37	40	63	61	47	43	32	21	5	34	48	47	39	43	26	18	5
32	39	42	65	63	49	44	35	25	6	36	52	50	40	44	29	20	6
33	42	45	69	66	51	49	39	28	8	39	53	54	42	48	32	24	6
34	44	47	70	68	52	51	42	31	9	41	55	58	43	49	34	26	7
35	46	49	71	69	53	53	44	33	9	43	57	62	46	50	37	29	9
36	48	51	75	70	55	56	50	37	10	45	58	62	49	53	39	29	12
37	50	54	75	71	58	58	53	38	14	47	61	64	50	56	40	31	12
38	53	56	77	72	62	62	55	40	14	49	62	66	52	59	44	34	13
39	55	58	78	73	65	65	58	43	15	51	62	68	55	62	46	34	13
40	57	61	80	74	69	67	60	46	18	53	63	69	56	63	48	36	16
41	59	63	82	78	71	70	61	47	20	55	65	71	59	65	49	39	20
42	61	64	82	79	74	70	63	50	21	57	68	72	61	66	52	41	24
43	63	66	84	79	74	72	64	53	22	59	71	74	63	69	55	43	29
44	64	67	84	80	76	72	65	56	22	61	74	76	64	69	56	45	29
45	65	68	85	81	76	74	67	58	24	62	76	77	65	69	58	46	29
46	67	70	86	83	77	75	69	60	27	63	78	77	67	72	59	48	29
47	68	71	87	84	78	76	69	62	28	65	78	77	69	75	61	51	29
48	70	72	89	86	79	78	71	63	29	67	79	79	73	75	63	54	29
49	71	74	90	87	80	79	72	64	31	68	81	79	73	75	64	56	31
50	72	75	91	88	80	80	73	67	36	69	83	81	74	76	65	59	32
51	73	76	92	89	81	81	74	68	38	70	83	82	75	77	67	60	33
52	74	77	93	90	81	81	75	69	39	71	83	84	76	77	68	63	35
53	75	78	94	90	82	81	77	71	41	72	84	84	77	78	69	64	36
54	76	79	94	91	83	83	78	72	44	74	85	85	78	80	70	66	38
55	77	80	94	92	84	84	78	73	45	75	89	86	78	82	71	67	39
56	78	81	94	93	85	85	78	74	46	76	91	86	79	84	73	68	41
57	80	82	95	94	85	86	79	75	51	78	91	87	81	84	74	70	46
58	81	83	96	94	87	87	80	76	53	79	91	88	83	85	75	71	48
59	82	84	97	94	88	88	80	77	54	80	92	89	83	87	76	73	51
60	83	85	97	96	89	89	81	79	56	81	92	90	84	88	78	73	54
61	84	86	97	97	89	89	82	81	60	82	92	91	85	89	78	75	56
62	85	87	97	97	90	90	83	81	65	84	93	91	85	90	81	76	61
63	86	88	97	97	90	91	85	83	66	84	93	92	87	90	81	78	62
64	86	88	97	97	90	91	85	84	66	85	93	92	89	91	81	80	63
65	87	89	98	97	91	92	86	86	68	86	93	93	91	91	82	81	64
66	88	89	98	98	91	92	87	87	69	87	94	94	91	92	84	82	65
67	89	91	98	98	91	93	88	89	74	88	96	94	92	93	86	83	65
68	90	91	98	98	92	93	89	89	77	89	97	94	93	93	88	84	67
69	91	92	98	98	93	94	89	90	80	90	97	95	93	93	90	86	68
70	92	93	98	98	93	94	91	92	84	91	97	96	93	93	90	88	70
71	92	94	98	98	94	94	91	93	84	91	98	96	93	94	92	89	70
72	93	94	98	98	94	95	93	94	84	92	98	96	95	95	93	90	73
73	94	95	98	98	94	96	93	96	86	93	98	96	96	95	93	93	74
74	94	95	98	98	94	96	93	97	86	94	98	96	96	96	93	94	77
75	95	96	98	98	95	97	94	98	86	94	98	97	96	96	95	94	79
76	95	96	98	99	96	97	94	98	87	95	98	97	96	97	95	94	82
77	96	96	98	99	96	97	95	99	88	95	99	97	96	97	96	94	83
78	96	97	98	99	96	97	95	100	89	96	99	97	96	98	96	95	85
79	96	97	98	99	96	97	95	100	90	96	99	98	97	98	97	96	86
80	97	97	98	99	96	97	96	100	91	97	99	98	98	98	97	97	88
81	97	97	98	99	97	97	96	100	93	97	99	98	98	98	98	98	88
82	98	98	98	99	97	97	97	100	95	97	99	99	98	98	98	98	88
83	98	97	98	99	97	97	97	100	96	98	99	99	99	99	98	98	92
84	98	97	98	100	97	97	97	100	98	98	99	99	99	99	98	99	94
85	99	98	98	100	97	98	97	100	98	99	100	99	99	99	98	99	95
86	99	99	99	100	97	98	98	100	99	99	100	100	99	99	99	99	96
87	99	99	99	100	98	98	99	100	99	99	100	100	100	99	100	99	97
88	99	99	99	100	98	99	99	100	99	99	100	100	100	99	100	99	97
89	99	99	99	100	98	99	99	100	99	99	100	100	100	100	100	99	97
90	99	99	99	100	98	99	99	100	99	100	100	100	100	100	100	99	98
91	99	99	100	100	98	99	100	100	99	100	100	100	100	100	100	99	98
92	100	99	100	100	99	99	100	100	99	100	100	100	100	100	100	99	99
93	100	99	100	100	99	99	100	100	99	100	100	100	100	100	100	100	99
94	100	100	100	100	99	99	100	100	99	100	100	100	100	100	100	100	99
95	100	100	100	100	99	99	100	100	99	100	100	100	100	100	100	100	99
96	100	100	100	100	99	99	100	100	99	100	100	100	100	100	100	100	99
97	100	100	100	100	99	100	100	100	99	100	100	100	100	100	100	100	99
98	100	100	100	100	99	100	100	100	99	100	100	100	100	100	100	100	99
99	100	100	100	100	99	100	100	100	99	100	100	100	100	100	100	100	99
100	100	100	100	100	100	100	100	100	100	100	100	100	100	100	100	100	100
M	**41.69**	**40.36**	**32.29**	**32.86**	**37.87**	**38.61**	**42.31**	**44.99**	**56.97**	**42.98**	**36.72**	**37.18**	**40.95**	**39.35**	**45.31**	**48.28**	**58.93**
SD	17.54	17.30	13.06	13.62	17.37	16.58	17.58	15.63	15.84	17.65	14.85	15.82	16.62	16.42	17.03	17.09	17.77
mdn	37	36	29	28	33	34	37	42.5	57	39	32	33	37	35	42	47	59
mode	20	20	20	20/21	20/25	24/33	26/36	21/32	67	20	20	24	28	28	24/30	24/33/50	57
Sum score	Total	Male								Female							
Age [yrs]	all	all	≤24	25-34	35-44	45-54	55-64	65-74	≥75	all	≤24	25-34	35-44	45-54	55-64	65-74	≥75
Note. Male Total n = 1227, Female Total n = 1273; sub-sample sizes see gender- and age-specific tables.																	
